# Supplementary material for: Mental search of concepts is supported by egocentric vector representations and restructured grid maps
Source: Nat Commun. 2023 Dec 8;14:8132. doi: 10.1038/s41467-023-43831-w (PMC10709434; doi:10.1038/s41467-023-43831-w)
Supplement: Supplementary file 1 — Supplementary Information [file 41467_2023_43831_MOESM1_ESM.pdf]

Mental search of concepts is supported by egocentric vector representations and restructured grid maps

Simone Viganò[1,2,†], Rena Bayramova[1], Christian F. Doeller[1,3,4*], Roberto Bottini[2,*]

[1] Max Planck Institute for Human Cognitive and Brain Sciences, Leipzig, Germany
[2] Center for Mind/Brain Sciences, University of Trento, Rovereto, Italy
[3] Kavli Institute for Systems Neuroscience, Centre for Neural Computation, The Egil and Pauline Braathen and Fred Kavli Centre for Cortical Microcircuits, Jebsen Centre for Alzheimer's Disease, Norwegian University of Science and Technology, Trondheim, Norway
[4] Wilhelm Wundt Institute of Psychology, Leipzig University, Leipzig, Germany.
[*] these authors jointly supervised this work
† corresponding author: S Viganò vigano@cbs.mpg.de

Supplementary Materials

Supplementary Figure 1
Supplementary Figure 2
Supplementary Figure 3
Supplementary Figure 4
Supplementary Figure 5
Supplementary Figure 6
Supplementary Figure 7
Supplementary Figure 8
Supplementary Figure 9
Supplementary Figure 10
Supplementary Figure 11
Supplementary Figure 12
Supplementary Figure 13
Supplementary Figure 14
Supplementary Figure 15
Supplementary Figure 16
Supplementary Table 1
Supplementary Table 2
Supplementary Table 3
Supplementary Video 1
Supplementary Video 2

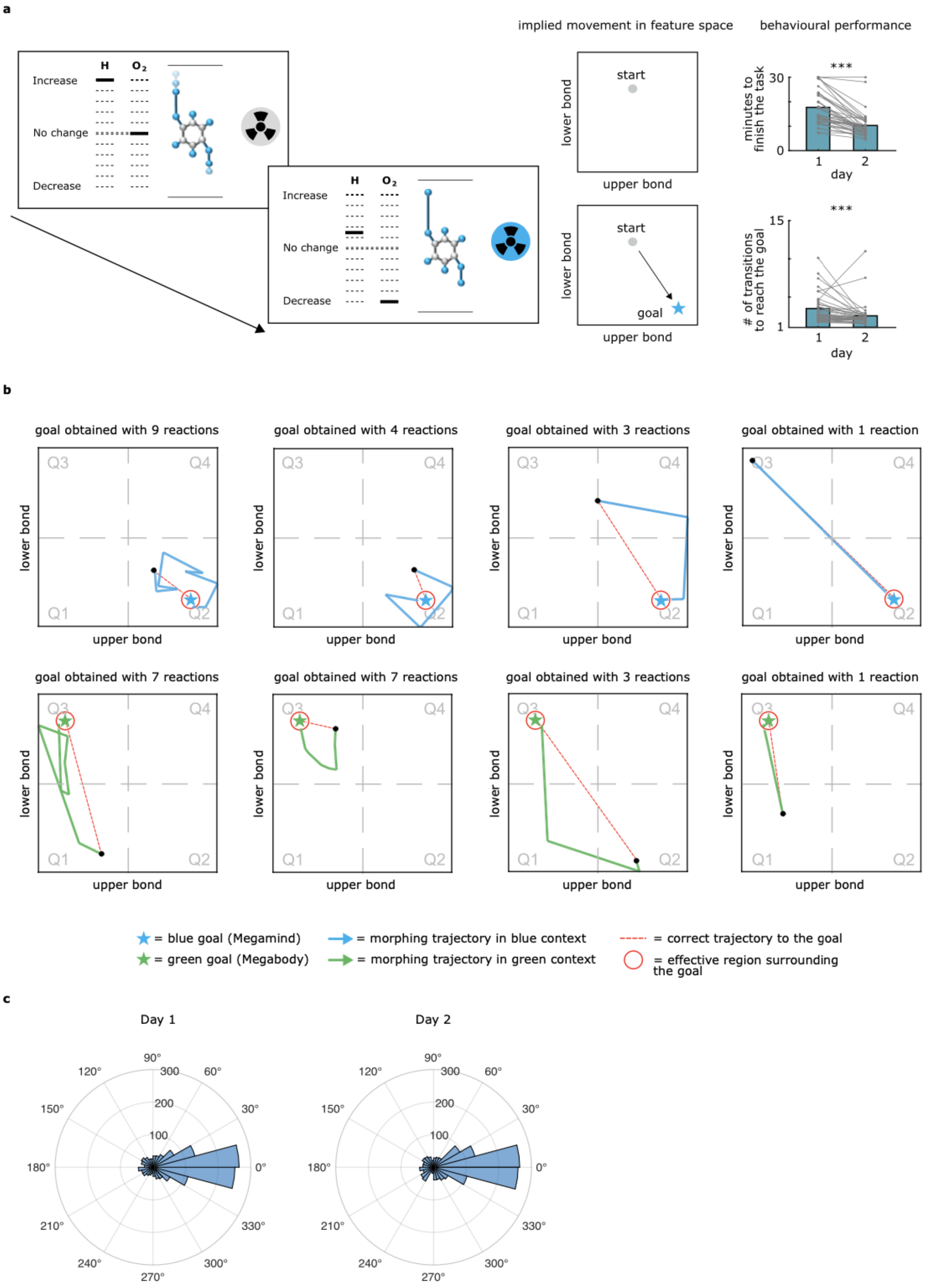

Supplementary Fig. 1 | Collect task with hint (training). a, During the first training task ("Collect task with hint", see Methods) participants actively modified the bond-length ratio of a wrong molecule to match the respective conceptual goal. Visual feedback (the hint) signaled when the correct configuration was reached and when the participant could "collect" the molecule to proceed to the next trial. Almost all of the participants (35/40, 87.5%) collected all the required molecules (20 blue, 20 green) within the time limit of 30 minutes on the first day already (mean time on day 1 = 17.7 min, std = 7.05 min) and significantly improved their performance on the next day, as

indicated both by the time taken to finish the task (mean time on day 2 = 10.3 min, std = 5.24 min; mean differential time day 2 - day 1 = -7.42 min, std = 5.43 min; z-score = -5.37, p = < 0.001, Wilcoxon sign rank test) and by the number of transitions necessary to reach the goal (mean number of transitions on day 1 = 3.49, std = 1.91; on day 2 = 2.55, std = 1.69; mean difference between days = -0.94, std = 2.18; z-score = -3.98, p = < 0.001, Wilcoxon sign rank test; perfect performance: 1 transition). **b,** Examples of the implied trajectories navigated by participants in the 2D feature spaces. On some trials (left) they tried many reactions/trajectories before finding the goal, while on other trials they reached it more directly (right). **c**, Distribution of all the first trajectories taken by participants during the Collect Task, expressed as error from the perfect trajectory to the goal. A value of 0° would indicate a perfect performance, a value of 180° would indicate navigation in the opposite direction.

a

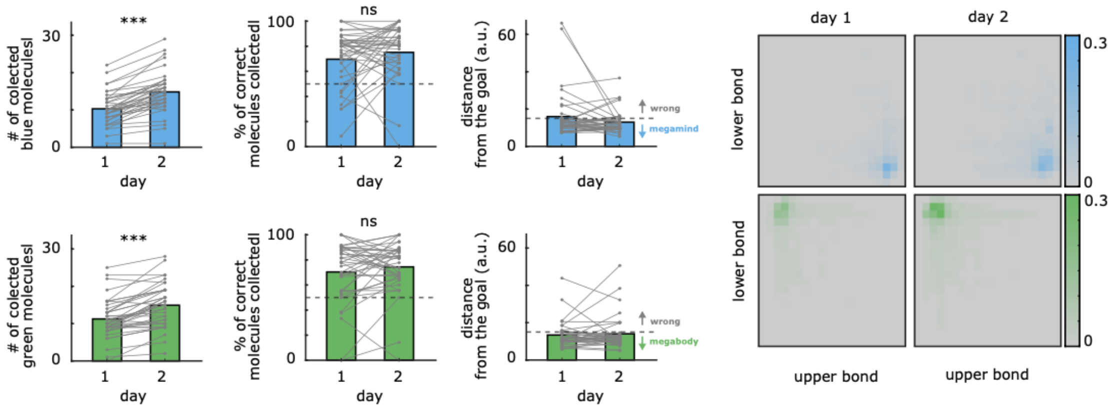

Supplementary Fig. 2 | Collect task without hint (training). a, During the second task (“Collect task without hint”, see Methods) participants followed the same procedure of the previous task but now i) they didn’t have any visual feedback, ii) they could also collect wrong molecules (that would count as mistakes), and iii) they had only 3 minutes per color context to collect as many correct molecules as they could. Results indicated that i) participants collected more molecules on day 2 compared to day 1 (mean increment blue molecule = 4.52 corresponding to 43.9% with respect to day 1 (std = 2.74); z-score = 5.37, $p < 0.001$, Wilcoxon sign rank test - mean increment green molecule = 3.68 corresponding to 32.7% with respect to day 1 (std = 3.14); z-score = 4.99, $p < 0.001$, Wilcoxon sign rank test), ii) the percentage of correct molecules was >=70% on both days ($p > .10$), and iii) that, on average, they collected molecules at short distances from the goal locations (mean distance from the goal on day 2 = 12.9 a.u. (std = 6.06) and 14.04 a.u. (std = 8.94) for the blue and green molecule respectively, with 32/40 participants (80%) having an average distance from the goal below the correct threshold of 15 a.u. for both molecules).

a

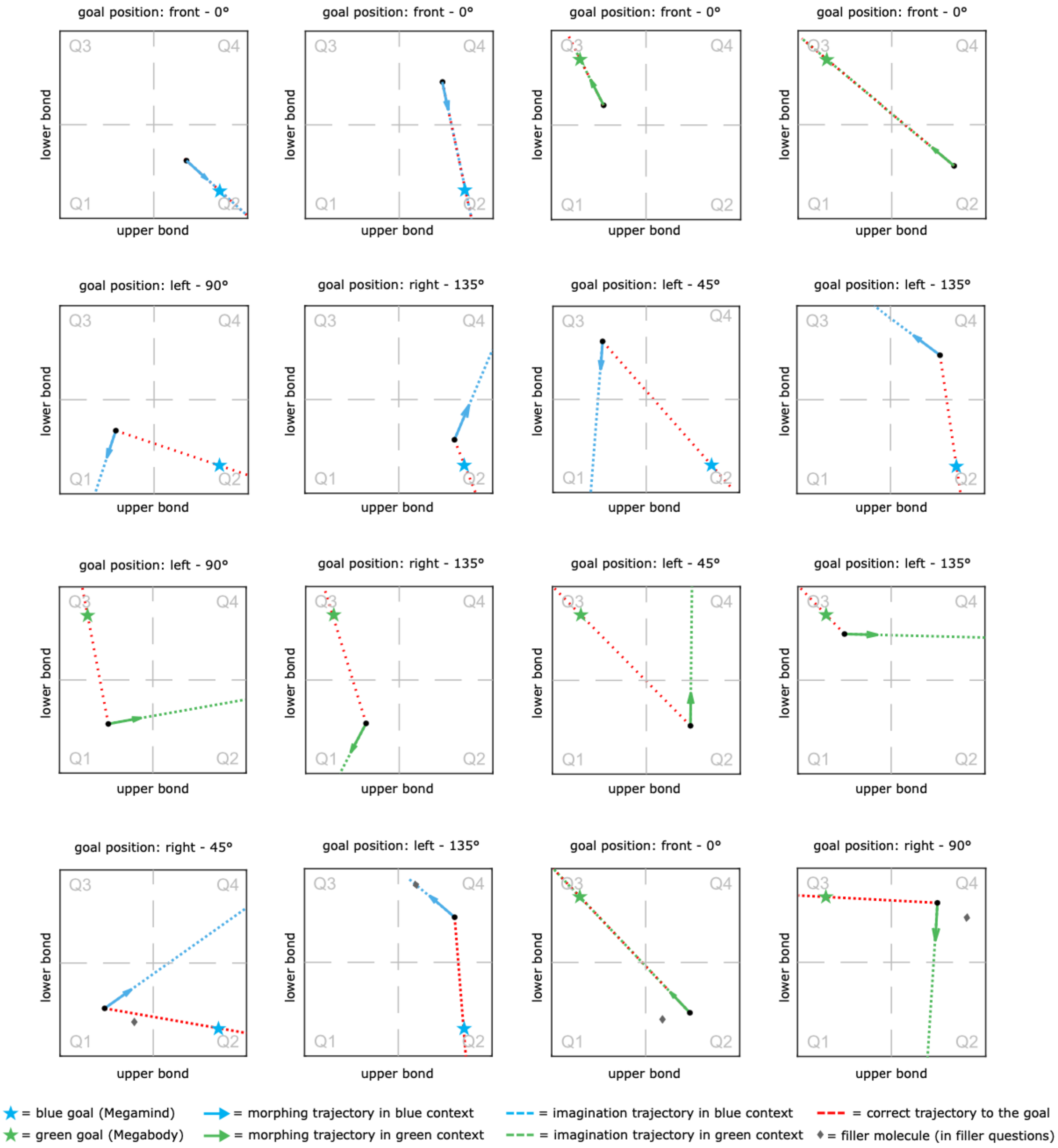
goal position: front - 0°
goal position: front - 0°
goal position: front - 0°
goal position: front - 0°
goal position: left - 90°
goal position: right - 135°
goal position: left - 45°
goal position: left - 135°
goal position: left - 90°
goal position: right - 135°
goal position: left - 45°
goal position: left - 135°
goal position: right - 45°
goal position: left - 135°
goal position: front - 0°
goal position: right - 90°
Q1
Q2
Q3
Q4
lower bond
upper bond
= blue goal (Megamind)
= green goal (Megabody)
= morphing trajectory in blue context
= morphing trajectory in green context
= imagination trajectory in blue context
= imagination trajectory in green context
= correct trajectory to the goal
= filler molecule (in filler questions)

b

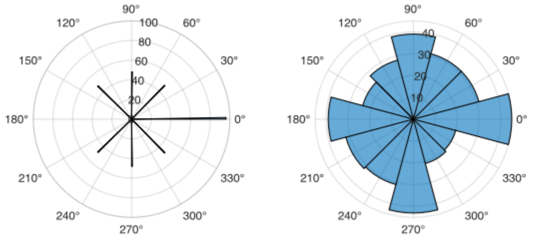
90°
120°
60°
150°
30°
180°
0°
210°
330°
240°
300°
270°
100
80
60
40
20
10
30

c

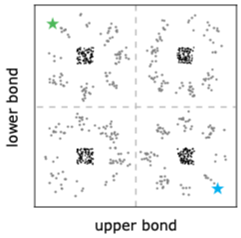
lower bond
upper bond

Supplementary Fig. 3 | Example trajectories in the Recall task (training + fMRI). a, Participants were presented with random wrong molecules that started morphing (solid arrow) and they were instructed to imagine the morphing to continue in the same way (blue or green dashed line). They were asked to indicate whether the morphing would result in the correct configuration or not: the correct trajectory that would lead to the goal is indicated as a red dashed line. When the blue/green and the red dashed lines overlap, it means that this is a trajectory directed to the goal (on-target trial) and participants are expected to respond “Yes”. When they do not overlap, they are expected to respond “No” (off-target trials). Off-target trials could miss the goal at various “egocentric-like conditions”: 135° to the left, 90° to the left, 45° to the left, 45° to the right, 90° to the right, 135° to the right (see Main Text and Methods). **b**, Distribution of the sampled directions for egocentric conditions (left) and allocentric conditions (right). Egocentric directions are referenced to the target direction to the goal (0°), while allocentric conditions are referenced to the horizontal axis (0°). **c,** distribution of starting (black) and ending (grey) coordinates for one participant.

a

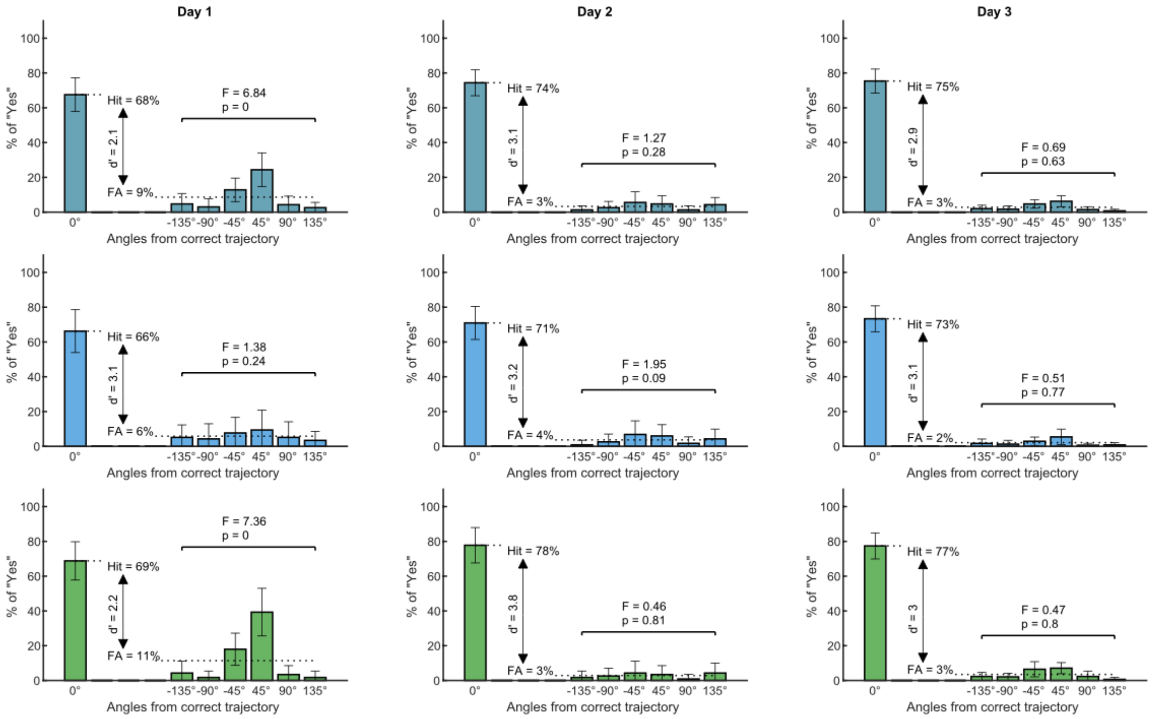

Supplementary Fig. 4 | Performance in the Recall task across the 3 days of experiment - Accuracy in the target question. a, For each day, we report the % of "Yes" responses as a function of the egocentric-like angular condition. 0° refers to on-target trials, and the % of Yes judgments here corresponds to the Hit rate. The average of the % of Yes judgments for the other conditions (-135°, -90°, etc) corresponds to the False Alarm (FA) rate. These two values are also used to compute a summary measure of the sensitivity to the correct trajectories (d'). An F-test is computed on the off-target conditions to verify whether any of them is significantly different from the others, which doesn't seem to be the case on day 3, during the fMRI scanning day. The first row indicates the performance when the two context/molecules are averaged, while the second and the third shows performance for the blue and green context, respectively. p = 0 indicates p <.001

a

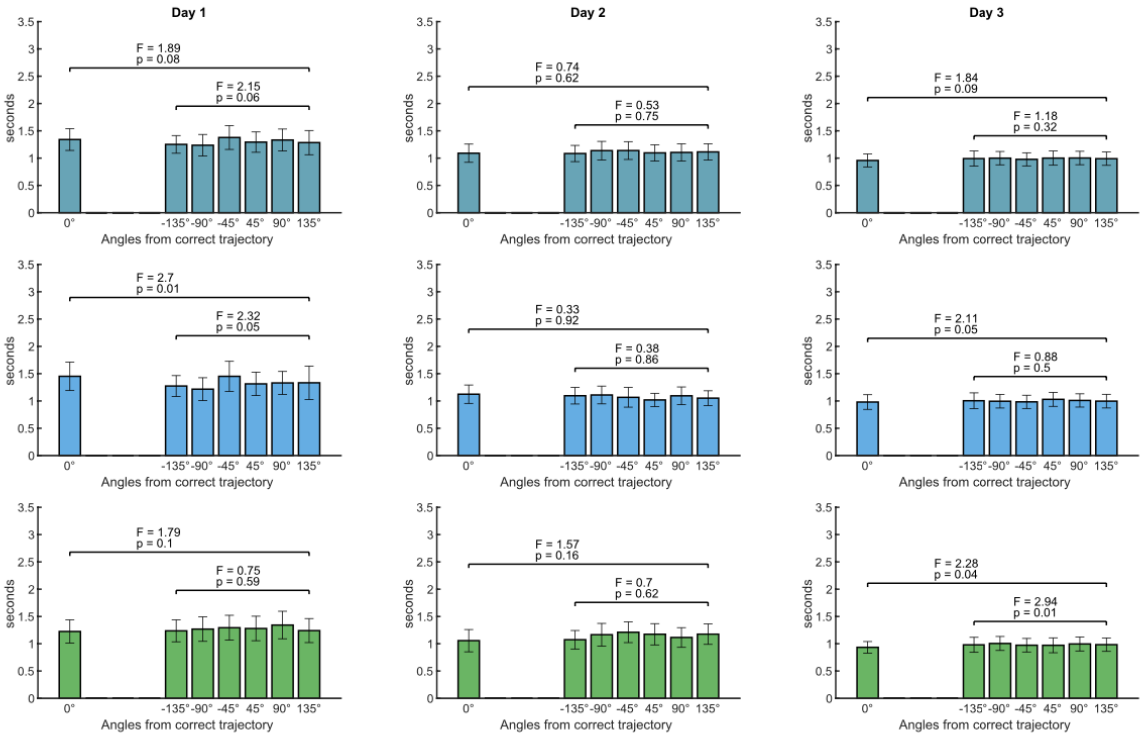

Supplementary Fig. 5 | Performance in the Recall task across the 3 days of experiment - Reaction Times in the target question. a, For each day, we report the Reaction Times (RTs) of the responses as a function of the egocentric-like angular condition. 0° refers to on-target trials. An F-test is computed both on all trials and on the off-target conditions solely to verify whether any of them is significantly different from the others. The first row indicates the performance when the two context/molecules are averaged, while the second and the third shows performance for the blue and green context, respectively.

a

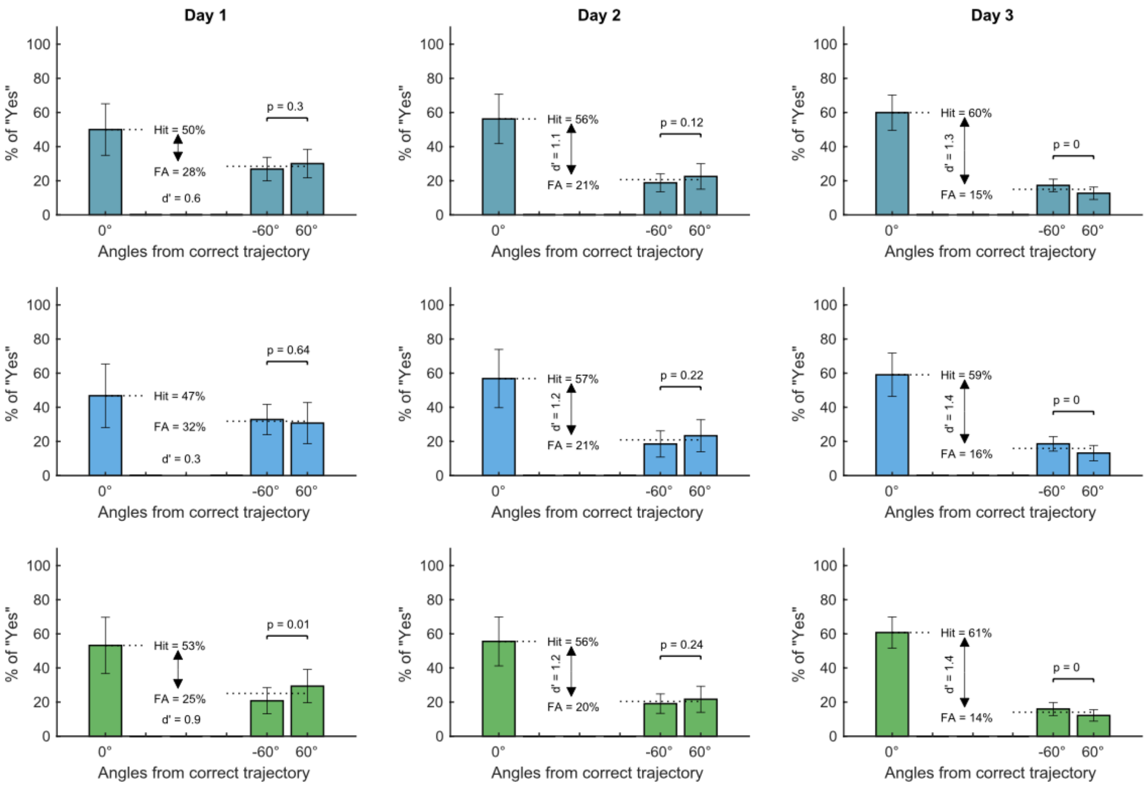

Supplementary Fig. 6 | Performance in the Recall task across the 3 days of experiment - Accuracy in the filler question. a, For each day, we report the % of “Yes” responses as a function of the angular trajectory from the filler goal (see Main text and Methods). 0° refers to on-target trials. The statistics reported correspond to those in Supplementary Fig. 4, adapted to the details of this question. We observed an interesting bias consisting of higher accuracy for “filler off-target trials” for one of the two off-target trajectories. This effect is unlikely to reflect an alteration of the global map and we cannot offer any conclusive interpretation of it, although it is worth noting that for one of the two contexts (green one, last row), this “side preference” was in the opposite direction on the first day, and it changed on the 3rd one to match the same direction of the blue context (second row). A potential interpretation is that this behavioral effect reflects the realignment of the two spaces into one via mental rotation, suggesting for instance that it was the green context, on average, that was rotated, but this remains highly speculative at the moment and should be taken into account with caution.

a

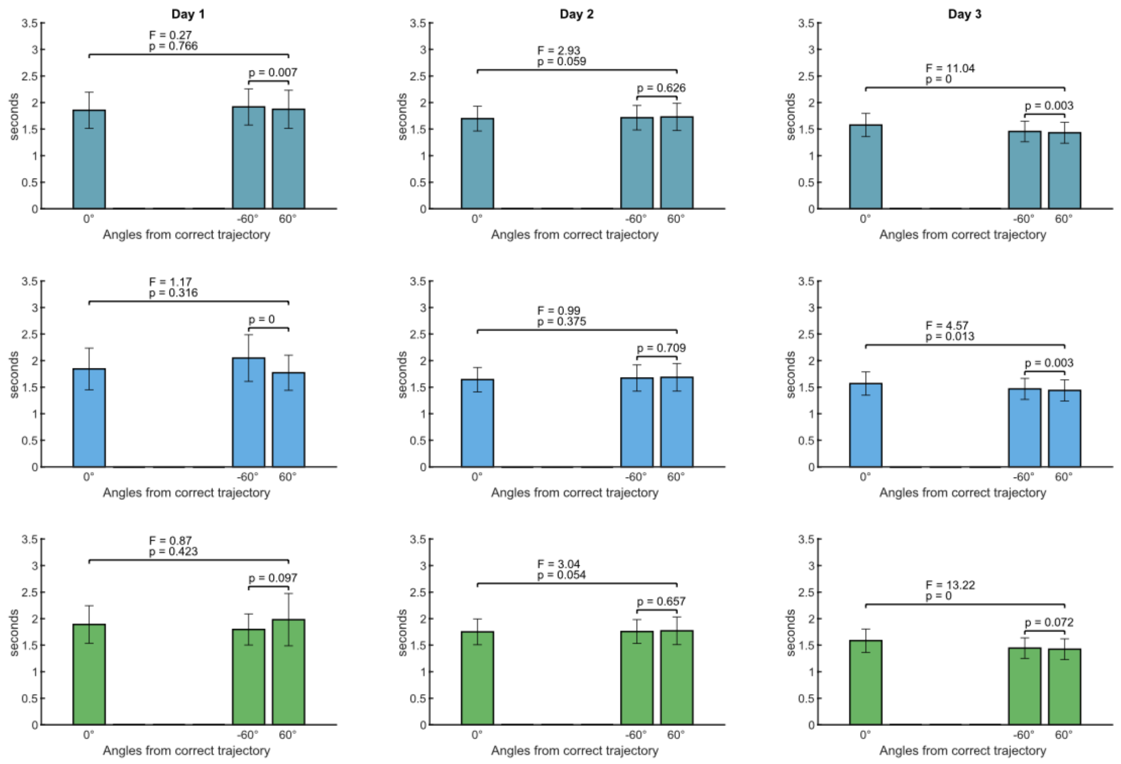

Supplementary Fig. 7 | Performance in the Recall task across the 3 days of experiment - Reaction Times in the filler question. a, For each day, we report the Reaction times (RTs) of the responses as a function of the angular trajectory from the filler goal (see Main text and Methods). 0° refers to “on-target filler trials”.

a

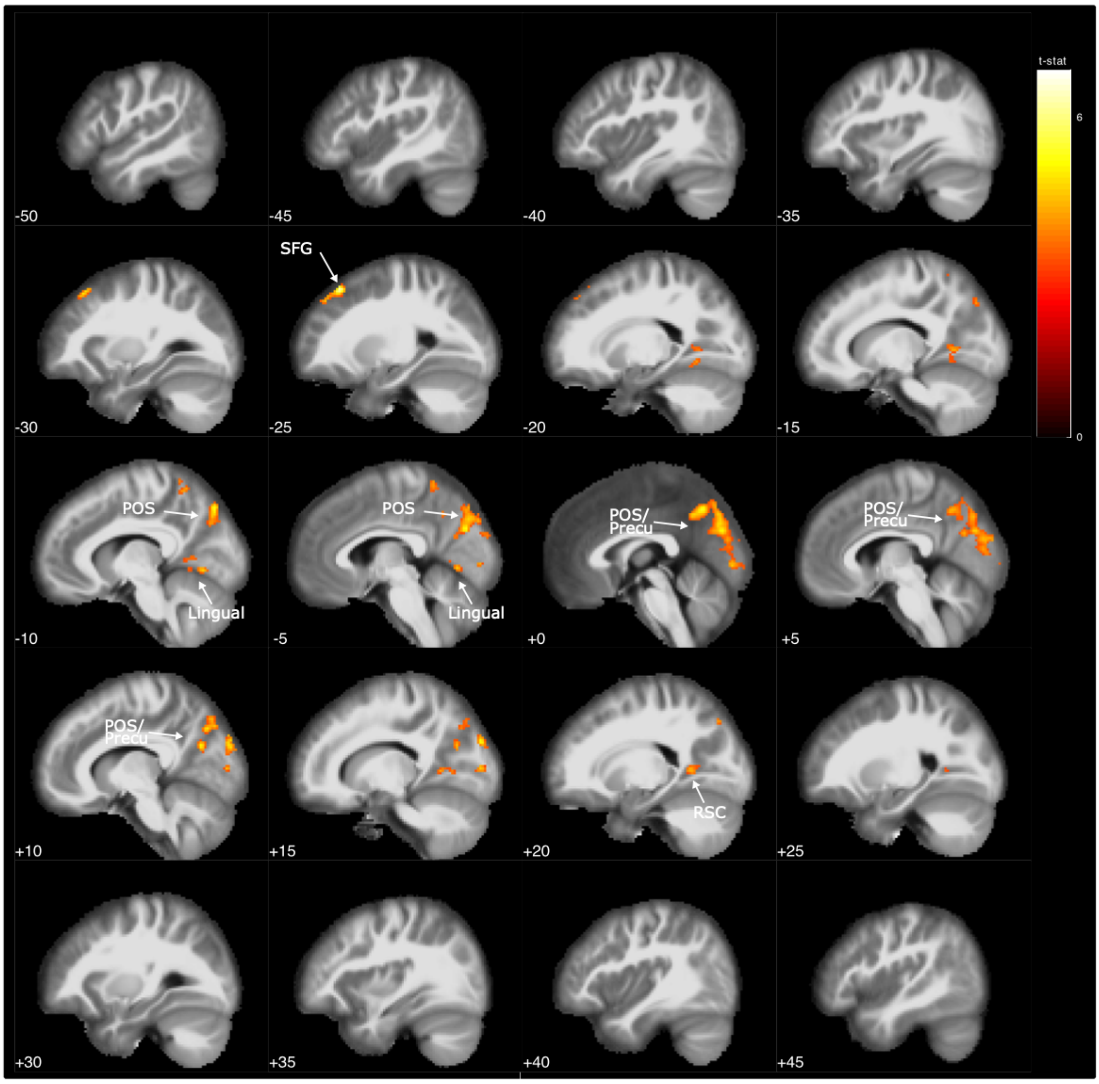

b

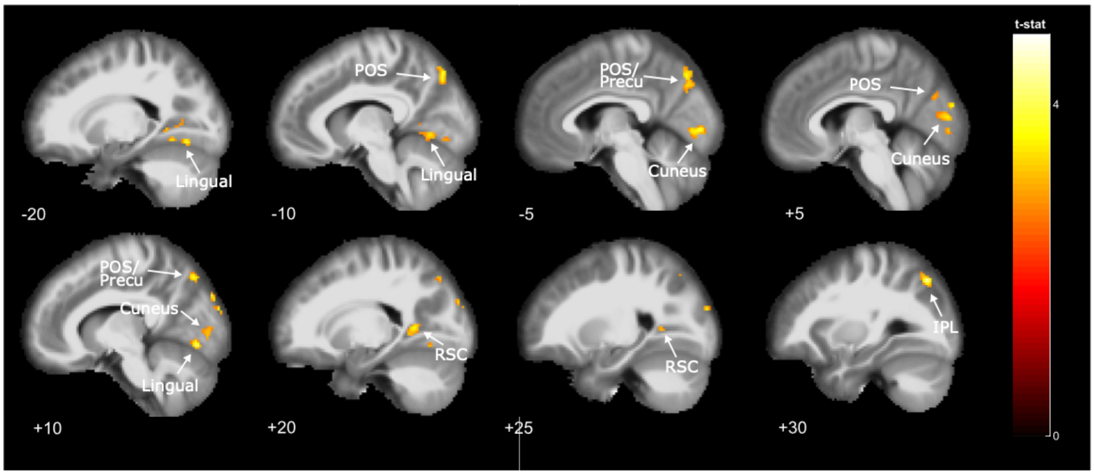

Supplementary Fig. 8 | Whole-brain adaptation effect of egocentric-like conditions with and without on-target trials. a, extensive whole-brain results for the analysis reported in Fig 2b-c. **b,** As a control, we repeated the main analysis reported in Fig. 2b-c but now focusing only on off-target trials, thus excluding those

trajectories that were directed to the goal, revealing that the effect was still present and thus it was not dependent on on-target trials. Statistical maps are thresholded at $p<.001$ at voxel level, and corrected for multiple comparisons at cluster level with $p<.05$. Significant clusters were found in parieto-occipital sulcus (-8 -78 44), lingual gyrus (16 -72 -8), cuneus (-4 -86 -2), retrosplenial cortex (16 -44 -2), and inferior parietal lobule (30 -72 48).

a

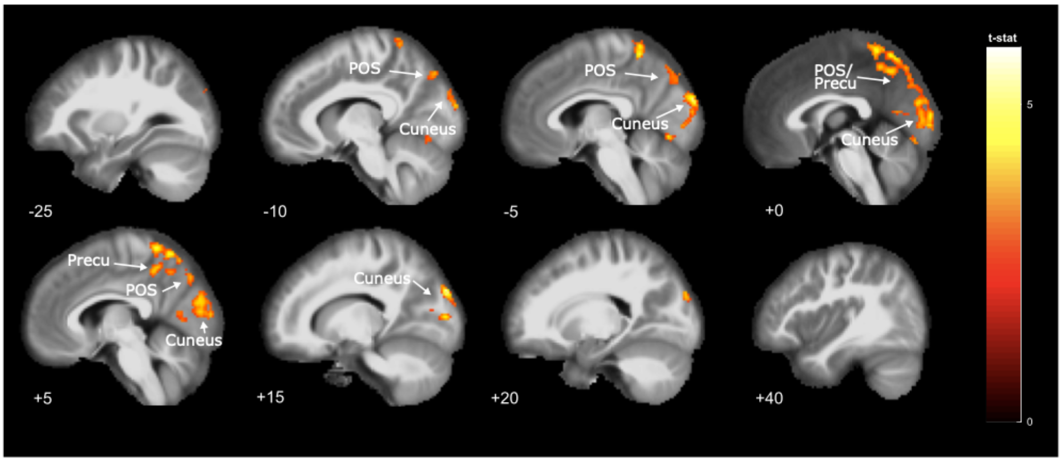

b

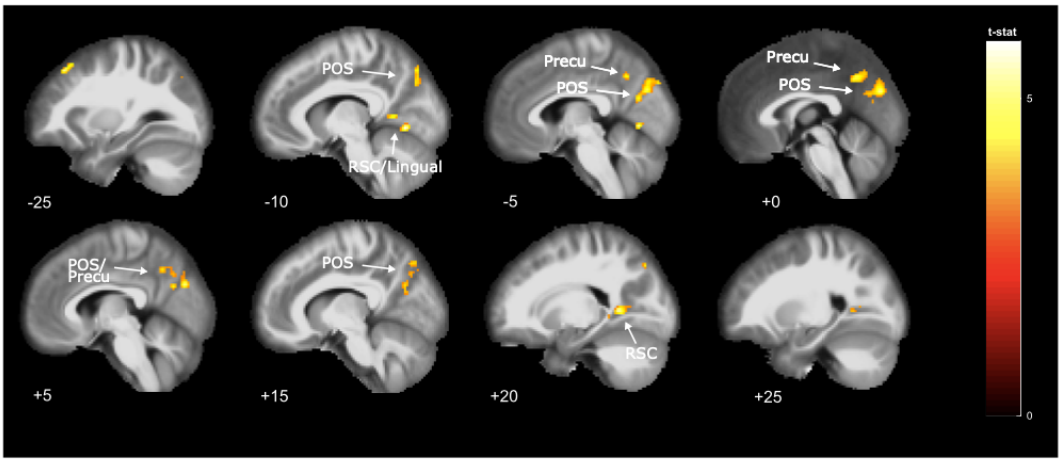

Supplementary Fig. 9 | Whole-brain adaptation effect of egocentric-like conditions for the two contexts separately. a, blue context; b, green context. As a control, we repeated the main analysis reported in Fig. 2b-c but now focusing on the two contexts separately, revealing that the effect was still present in the medial parietal cortex and thus it was not dependent on one specific molecule. Nevertheless, evident differences were observable in the specific and precise anatomical positions of some of the clusters. A potential interpretation, in light of the mental rotation analysis (see Results and Discussion) is that participants were maintaining one of the two contexts as reference and were rotating, or aligning, the second, thus recruiting additional brain regions. At the present moment this remains an interpretation, which would need further experiments to be properly clarified. Statistical maps are thresholded at $p<.001$ at voxel level, and corrected for multiple comparisons at cluster level with $p<.05$. Significant clusters were found in the cuneus (14 -92 30) and precuneus/PCC (-4 -50 70 and 2 -50 52) for the blue context, and in the POS (-4 -78 30), retrosplenial cortex (-10 -58 6), superior frontal gyrus (-28 30 52), lingual gyrus (-8 -70 -4) for the green context.

a

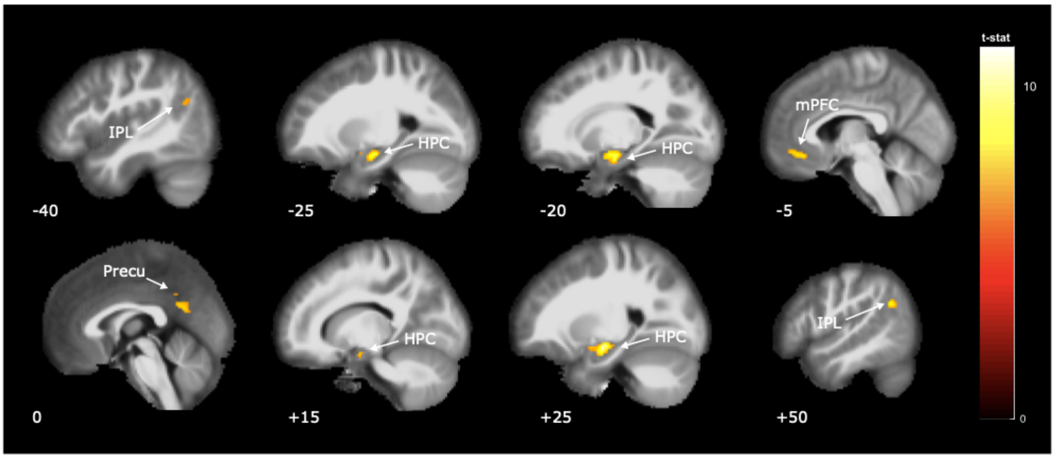

b

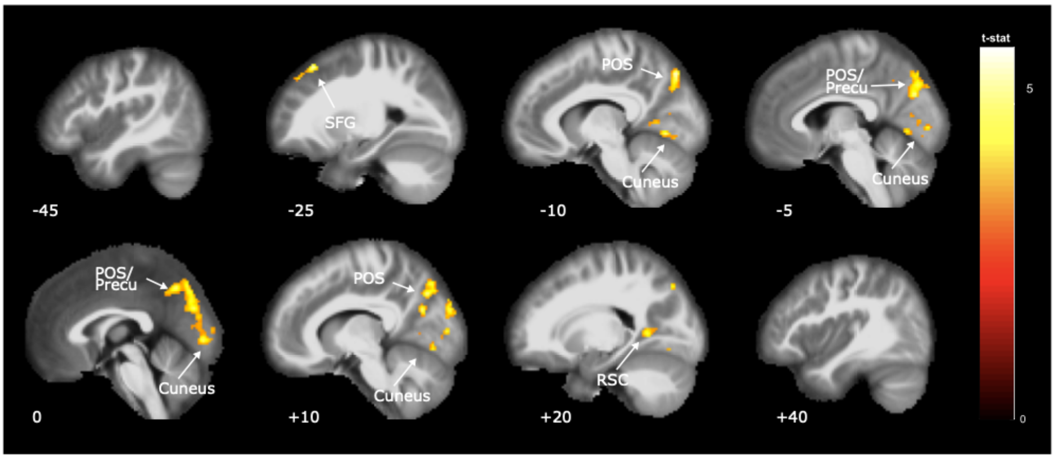

Supplementary Fig. 10 | Whole-brain adaptation effect of egocentric-like conditions after controlling for distance. As a control analysis, we repeated the main analysis reported in Fig. 2b-c after controlling for the distance to the goal. We reasoned that egocentric-like conditions, in principle, convey information about the relative distance to the goal, namely whether (and how much) we are getting closer to the goal or not. We created an additional parametric modulator modeling this information for each trial, which, across runs and subjects, was poorly correlated with our main regressor for egocentric-like conditions (Pearson's r = 0.10). Nevertheless, to provide conclusive evidence that the egocentric-like effect was independent of the distance effect, we put both the parametric modulators in the same GLM, looking for adaptation to the egocentric-like conditions after controlling/excluding the brain activity modulated by distance. Whole-brain results for the distance modulator and the controlled egocentric-like modulator are reported in **a** and **b**, respectively. Statistical maps are thresholded at $p<.001$ at voxel level, and corrected for multiple comparisons at cluster level with $p<.05$. Significant clusters were found in the bilateral hippocampi (24 -12 -16 and -24 -14 -18), bilateral inferior parietal lobules (48 -58 30 and -44 -64 24), precuneus (0 -58 24) and medial prefrontal cortex (-6 36 -14) for distance effect, and in the POS/precuneus (-12 -80 48), superior frontal gyrus (-28 28 54), cuneus (8 -78 6) and bilateral retrosplenial cortices/lingua gyrus (-18 -60 2 and 20 -54 4) for the egocentric effect.

a

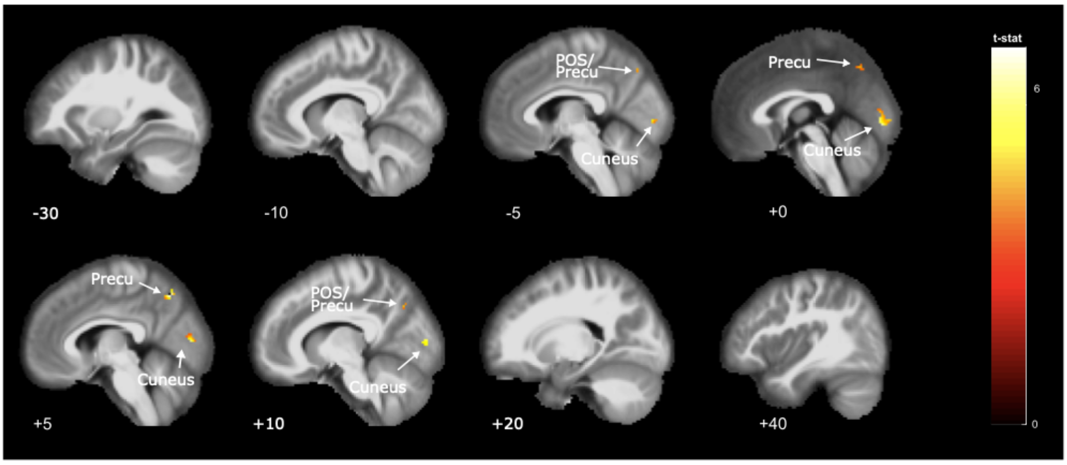

Supplementary Fig. 11 | Whole-brain adaptation effect of “left” vs “right” egocentric-like conditions. a, As a control, we repeated the main analysis reported in Fig. 2b-c but now modeling the (log) time since the last presentation of a trajectory that had the goal on one side (left or right), irrespective of the magnitude of the angle (if 45°, 90°, or 135°). Results indicate a significant cluster in the POS territory ($p<.001$ corrected at cluster level), although an ROI control reveals that this effect was significantly stronger in our main analysis (mean difference with the main effect = 0.37, std = 0.92, z-score = 2.5, $p < 0.05$, Wilcoxon sign rank test), when we modeled both the side (left vs right) and the angular magnitude (45°, 90°, 135°), suggesting that this brain region is associated with a fine-graded code of egocentric-like conditions. Statistical maps are thresholded at $p<.001$ at voxel level, and corrected for multiple comparisons at cluster level with $p<.05$. Significant clusters were found in the precuneus (4 -66 52) and in the cuneus (14 -86 4).

a

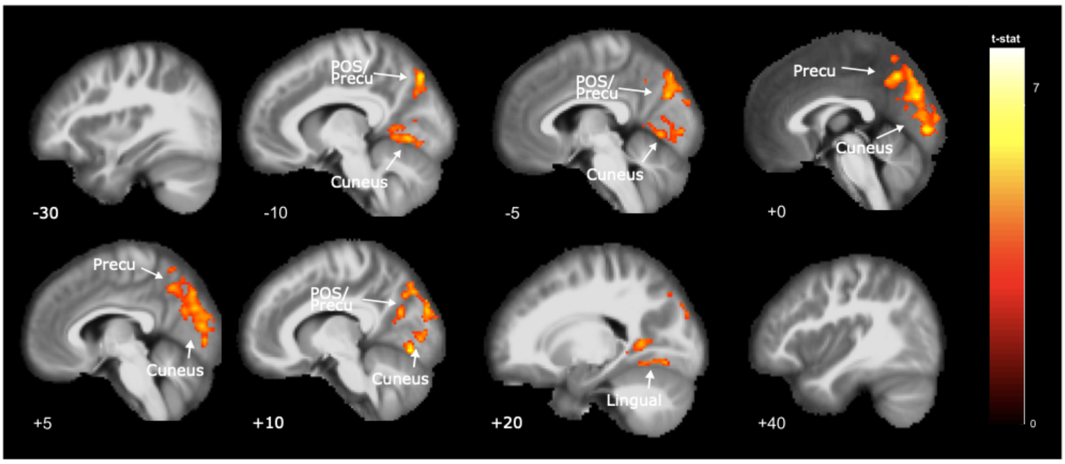

Supplementary Fig. 12 | Whole-brain adaptation effect of egocentric-like conditions after controlling for starting position. Starting positions were selected pseudo-randomly across the experiment, being balanced across the four quadrants. This makes it unlikely that our main effect of egocentric-like responses could be explained by repetition of the starting position. Nevertheless, as a further control, we ran an additional analysis to verify that this was the case: our main regressor of interest modeling egocentric conditions was put after a control regressor modeling the (log) time since the last presentation of a trajectory that started in a specific quadrant. Whole-brain analysis revealed very similar results to our main analysis after controlling for and excluding the variance explained by this potentially confounding variable (that is, working on its residuals). Statistical maps are thresholded at p<.001 at voxel level, and corrected for multiple comparisons at cluster level with p<.05. Significant clusters were found in the cuneus (0 -88 -2), retrosplenial cortex/lingual gyrus (16 -56 4), POS/precuneus (-4 -78 32), precuneus (-8 -54 64).

a

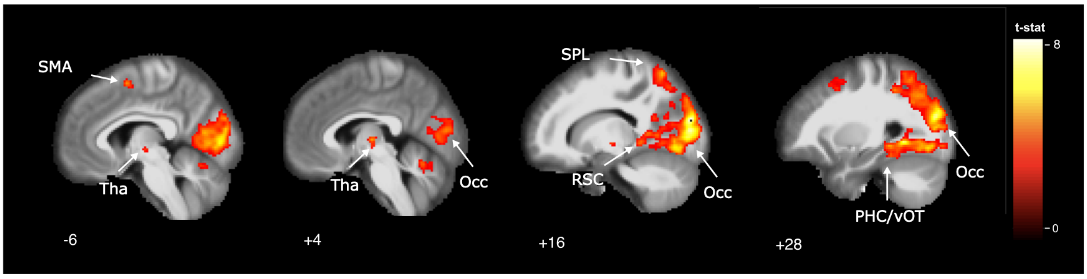

b

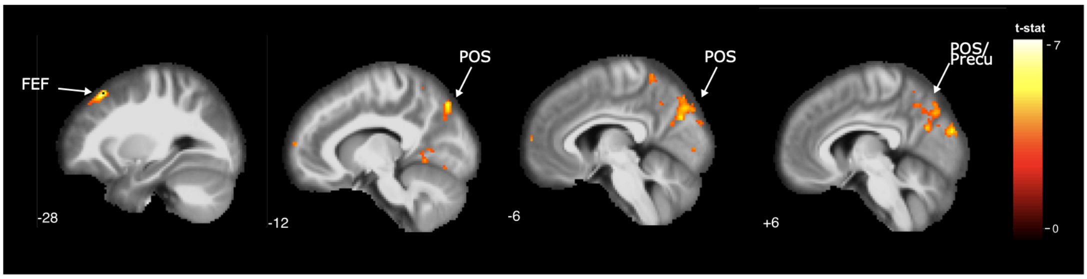

Supplementary Fig. 13 | Whole-brain adaptation effect of egocentric-like conditions after controlling for allocentric head-direction like conditions. a, Allocentric directions were resampled with a granularity of 30°, to allow a proper implementation of the time-dependent adaptation analysis and for consistency with the grid-like analysis (see Figure 4). The resulting clusters reveal similarities with previous studies showing allocentric head-direction like signals in the human brain during virtual reality fMRI experiment e.g., Doeller et al. 2010 and Shine et al. 2015, specially in the RSC and the Thalamus (Tha). b, adaptation of egocentric like conditions after controlling for allocentric effects. Statistical maps are thresholded at $p<.001$ at voxel level, and corrected for multiple comparisons at cluster level with $p<.05$. Significant clusters were found in the occipital lobe (16 -90 18), thalamus (14 -14 2), parahippocampus (32 -26 -20) for the allocentric “head direction like” effect, and in the superior frontal gyrus/frontal eye fields (-28 28 54), precuneus/POS (-12 -80 48), and retrosplenial cortex (-18 60 2) for the egocentric effect.

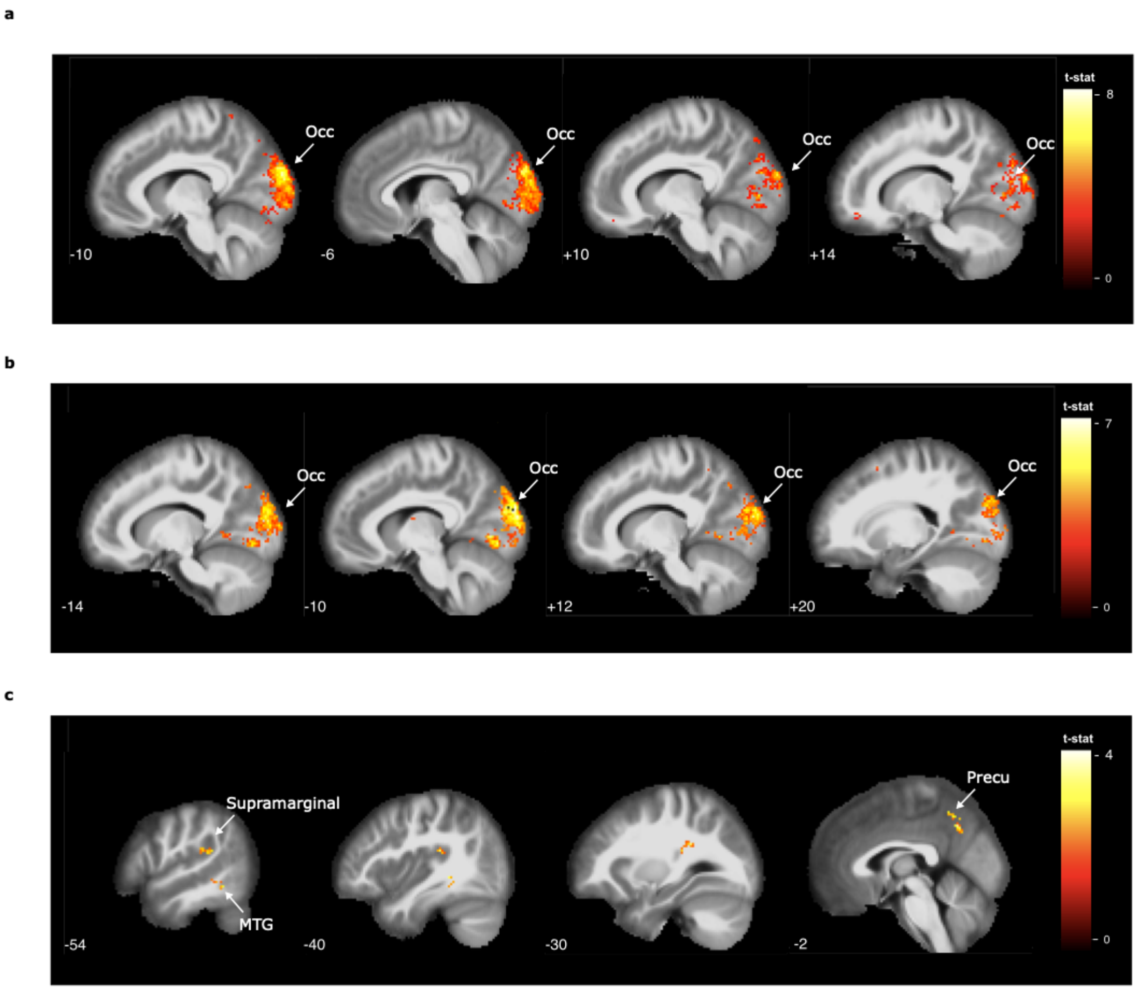

Supplementary Fig. 14 | Whole brain decoding of egocentric conditions within contexts over the cross-context decoding a-b, within context decoding of egocentric conditions reveals significantly stronger clusters in the occipital cortex compared to the cross context decoding. Statistical maps are thresholded at p<.001 at voxel level, and corrected for multiple comparisons at cluster level with p<.05. Significantly stronger clusters were found in the occipital cortex when we compared within context vs cross context decoding (-8 -94 26 and -10 -96 22 respectively for blue>cross and green>cross). **c**, Significantly stronger clusters were found in the precuneus (-2 -60 42), supramarginal gyrus (-48 -32 22), and MTG (-40 -40 -4) when we compared cross context decoding vs blue context decoding with a threshold of p<.01 at voxel level, corrected for multiple comparisons at voxel level with p<.05.. No statistically significant clusters were obtained for the contrast cross context decoding vs green context decoding.

a

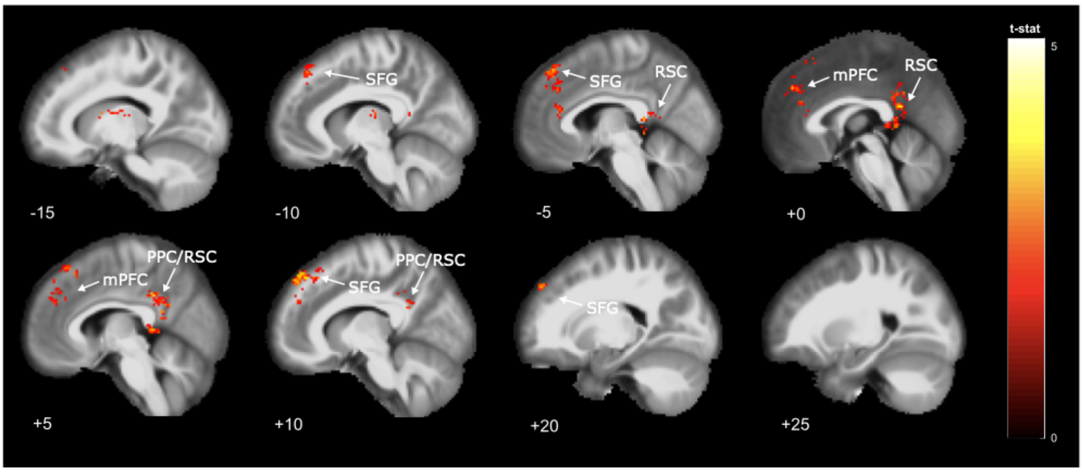

Supplementary Fig. 15 | Whole-brain decoding analysis without on-target trials. a, As a control analysis, we repeated the main analysis reported in Fig. 2d-e but now focusing only on off-target trials, thus excluding those trajectories that were directed to the goal, revealing that the effect was still present, although weaker, and thus it was not completely dependent on on-target trials. Statistical maps are thresholded at p<.05 at voxel level, and corrected for multiple comparisons at cluster level with p<.05. Significant clusters were obtained in the superior frontal gyrus/frontal eye field (14 48 44) and posterior parietal cortex extending in the retrosplenial cortex (2 -48 20).

a

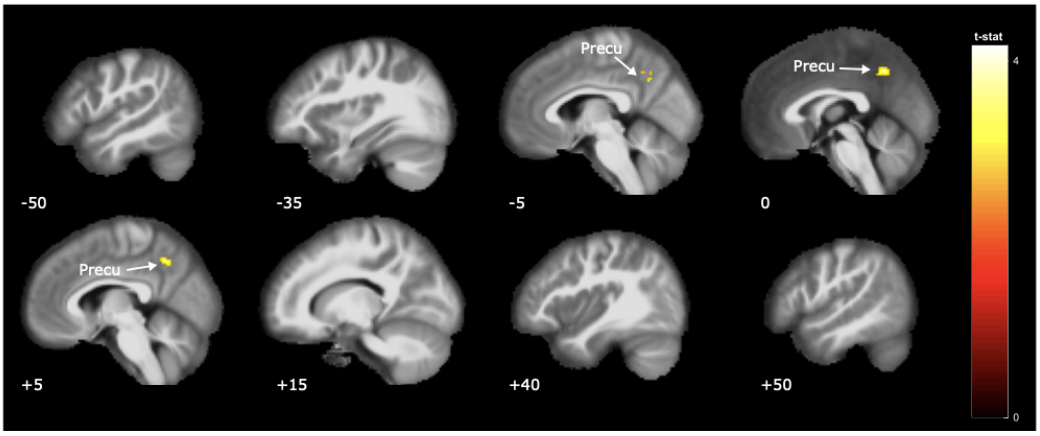

Supplementary Fig. 16 | Whole-brain representational similarity analysis for mental rotation without goal quadrants. a, As a control analysis, we repeated the main analysis reported in Fig. 3a-b but now focusing only on quadrants that did not contain the goal, thus excluding Q2-blue and Q3-green. Results revealed that the effect in precuneus (-2 -56 44) was still significant ($p<.005$ at voxel level, corrected for multiple comparisons at cluster level with $p<.05$), thus indicating that this region was not simply representing cross-context correspondence of goal quadrants.

Region	Extent (voxels)	p (corr.)	p (uncorr.)	t-stat	x	y	z
Medial Occipito-Parietal Cortex/POS	1539	<.001	<.001	5.83	6	-92	22
	1539		<.001	5.68	-12	-80	48
	1539		<.001	5.67	-4	-76	30
Lingual gyrus	168	<.001	<.001	5.61	-8	-70	-4
Superior frontal gyrus/FEF	126	<.001	<.001	6.89	-28	28	54
Cuneus	83	0.001	<.001	4.29	20	-54	4
Precuneus	73	0.002	<.001	4.27	-8	-54	64

Supplementary Table 1 | Cluster (and sub-cluster) peaks of the whole-brain adaptation analysis for egocentric-like conditions reported in Fig. 2c. p (uncorr.) = uncorreced p-value at voxel level; p (corr.) = p-value corrected for multiple comparisons at cluster level; x,y,z coordinates are in MNI space. one-sided t-test as implemented in SPM12

Region	Extent (voxels)	p (corr.)	p (uncorr.)	t-stat	x	y	z
Right Inferior Parietal Lobule	92	0.003	<.001	4.9	36	-56	42
Precuneus	72	0.005	<.001	4.51	-2	-56	46
Left Inferior Parietal Lobule	69	0.005	<.001	4.93	-32	-62	40

Supplementary Table 2 | Cluster peaks of the whole-brain quadrant-by-quadrant similarity analysis (rotation effect) reported in Fig. 3b. p (uncorr.) = uncorreced p-value at voxel level; p (corr.) = p-value corrected for multiple comparisons at cluster level; x,y,z coordinates are in MNI space. one-sided t-test as implemented in SPM12

Region	Extent (voxels)	p (corr.)	p (uncorr.)	t-stat	x	y	z
Superior Parietal Lobule	293	<.001	<.001	5.18	-30	-46	66
Medial Prefrontal Cortex	112	0.001	<.001	4.46	2	48	-2
Left Angular Gyrus	75	0.004	<.001	4.19	-50	-70	18
Entorhinal Cortex	8	0.01	<.001	4.23	16	-6	-26

Supplementary Table 3 | Cluster peaks of the whole-brain analysis on the alteration of the grid-like signal reported in Fig. 4g. p (uncorr.) = uncorreced p-value at voxel level; p (corr.) = p-value corrected for multiple comparisons at cluster level; x,y,z coordinates are in MNI space. one-sided t-test as implemented in SPM12
