# Supplementary material for: Mental search of concepts is supported by egocentric vector representations and restructured grid maps
Source: Nat Commun. 2023 Dec 8;14:8132. doi: 10.1038/s41467-023-43831-w (PMC10709434; doi:10.1038/s41467-023-43831-w)
Supplement: Supplementary file 3 — Description of Additional Supplementary Files [file 41467_2023_43831_MOESM3_ESM.pdf]

Description of Additional Supplementary Files

File Name: Supplementary Movie 1
Description: Collect task: example trials of the collect task, where participants were asked to morph ineffective molecules until they matched the correct (effective) configurations.

File Name: Supplementary Movie 2
Description: Recall task: example trials for the recall task, where participants were asked to look at an ineffective molecule morphing for 1 second, then to imagine the morphing to continue for 4 seconds and to answer to the question of whether or not the morphing will ever result in the correct configuration.
