# Supplementary material for: Mental search of concepts is supported by egocentric vector representations and restructured grid maps
Source: Nat Commun. 2023 Dec 8;14:8132. doi: 10.1038/s41467-023-43831-w (PMC10709434; doi:10.1038/s41467-023-43831-w)
Supplement: Supplementary file 6 — Reporting Summary [file 41467_2023_43831_MOESM6_ESM.pdf]

nature portfolio

Corresponding author(s): Simone Viganò

Last updated by author(s): Oct 22, 2023

Reporting Summary

Nature Portfolio wishes to improve the reproducibility of the work that we publish. This form provides structure for consistency and transparency in reporting. For further information on Nature Portfolio policies, see our Editorial Policies and the Editorial Policy Checklist.

Statistics

For all statistical analyses, confirm that the following items are present in the figure legend, table legend, main text, or Methods section.

n/a	Confirmed	
☐	☒	The exact sample size (*n*) for each experimental group/condition, given as a discrete number and unit of measurement
☐	☒	A statement on whether measurements were taken from distinct samples or whether the same sample was measured repeatedly
☐	☒	The statistical test(s) used AND whether they are one- or two-sided<br>*Only common tests should be described solely by name; describe more complex techniques in the Methods section.*
☐	☒	A description of all covariates tested
☐	☒	A description of any assumptions or corrections, such as tests of normality and adjustment for multiple comparisons
☐	☒	A full description of the statistical parameters including central tendency (e.g. means) or other basic estimates (e.g. regression coefficient) AND variation (e.g. standard deviation) or associated estimates of uncertainty (e.g. confidence intervals)
☐	☒	For null hypothesis testing, the test statistic (e.g. *F*, *t*, *r*) with confidence intervals, effect sizes, degrees of freedom and *P* value noted<br>*Give P values as exact values whenever suitable.*
☒	☐	For Bayesian analysis, information on the choice of priors and Markov chain Monte Carlo settings
☐	☒	For hierarchical and complex designs, identification of the appropriate level for tests and full reporting of outcomes
☐	☒	Estimates of effect sizes (e.g. Cohen's *d*, Pearson's *r*), indicating how they were calculated

Our web collection on statistics for biologists contains articles on many of the points above.

Software and code

Policy information about availability of computer code

Data collection	MATLAB 2019b, Psychtoolbox 3.0.8
Data analysis	SPM12, CosmoMVPa

For manuscripts utilizing custom algorithms or software that are central to the research but not yet described in published literature, software must be made available to editors and reviewers. We strongly encourage code deposition in a community repository (e.g. GitHub). See the Nature Portfolio guidelines for submitting code & software for further information.

Data

Policy information about availability of data

All manuscripts must include a data availability statement. This statement should provide the following information, where applicable:

- Accession codes, unique identifiers, or web links for publicly available datasets
- A description of any restrictions on data availability
- For clinical datasets or third party data, please ensure that the statement adheres to our policy

The source data for the main findings generated in this study have been deposited in the OSF database without restrictions [https://doi.org/10.17605/OSF.IO/QTHVW].

Research involving human participants, their data, or biological material

Policy information about studies with human participants or human data. See also policy information about sex, gender (identity/presentation), and sexual orientation and race, ethnicity and racism.

Reporting on sex and gender	40 subjects in total, 19 selfi-identifying as "female" and 21 as "male". Our study did not address any gender- nor biological sex-related question.
Reporting on race, ethnicity, or other socially relevant groupings	We did not address any question related to ethnicity, race, or other socially relevant groupings, therefore this information was not collected from participants. Similarly, we did not collect genotypic information. All the participants reported no previous or current diagnosis of neurological or psychiatric disorders.
Population characteristics	mean age = 27.2 years (std = 4.8)
Recruitment	via Max Planck internal database (contacted via email)
Ethics oversight	The study was approved by the local Ethics Committee of Leipzig University, Germany (protocol number 159/21-ek).

Note that full information on the approval of the study protocol must also be provided in the manuscript.

Field-specific reporting

Please select the one below that is the best fit for your research. If you are not sure, read the appropriate sections before making your selection.

☒ Life sciences ☐ Behavioural & social sciences ☐ Ecological, evolutionary & environmental sciences

For a reference copy of the document with all sections, see nature.com/documents/nr-reporting-summary-flat.pdf

Life sciences study design

All studies must disclose on these points even when the disclosure is negative.

Sample size	Sample size was determined without an priori power analysis, due to the small number of experiments conducted in conceptual navigation and no existing research testing specifically the egocentric code in concept spaces. Moreover, the study involved several different types of analysis (fMRI adaptation, multivariate decoding, grid-like analyses), in addition to behavioral analyses, which further complicated the determination of an effect size of reference in previous literature. In an attempt to decide the relatively appropriate sample size, we took guidance from studies in this area which have drawn informative results from smaller pools of participants. For example, in studying goal direction signals in a spatial task, Chadwick and colleagues (2015) included 16 adult participants. In a study that was very influential in the development of our own (but that focused on allocentric coding schemes), 21 participants performed similar tasks 12. Based on these considerations, we refrained from forming precisely quantified expectations and aimed to test 40 participants, which was roughly twice the number tested in the majority of experiments in this area that consistently showed similar results for allocentric and egocentric navigation.
Data exclusions	We didn't exclude any participant
Replication	No replication was attempted
Randomization	The study did not include between-group analyses, thus no randomization was performed
Blinding	The study did not include between-group analyses, thus no blind assignment was performed

Reporting for specific materials, systems and methods

We require information from authors about some types of materials, experimental systems and methods used in many studies. Here, indicate whether each material, system or method listed is relevant to your study. If you are not sure if a list item applies to your research, read the appropriate section before selecting a response.

Materials & experimental systems

n/a	Involved in the study
☒	☐ Antibodies
☒	☐ Eukaryotic cell lines
☒	☐ Palaeontology and archaeology
☒	☐ Animals and other organisms
☒	☐ Clinical data
☒	☐ Dual use research of concern
☒	☐ Plants

Methods

n/a	Involved in the study
☒	☐ ChIP-seq
☒	☐ Flow cytometry
☐	☒ MRI-based neuroimaging

Plants

Seed stocks: na

Novel plant genotypes: na

Authentication: na

Magnetic resonance imaging

Experimental design

Design type: event related

Design specifications: 8 runs. There were 48 trials per run, divided in the following way. Irrespective of the question asked, morphing trajectories were directed to the goal (Megamind or Megabody, catch trials) 25% of the time (12/48 trials). The remaining 75% of trials (36/48) was equally divided into 6 egocentric-like conditions, missing the goal with angular distances to the correct trajectory of -135°, -90°, -45°, +45°, +90°, +135°. The sign (+ or -, arbitrarily defined) of these angular trajectories determined whether the goal was missed on the "left" or on the "right". As the target question happened on 50% of the trials, participants were expected to answer Yes to this question on 6 trials, and No on 18, for a total of 24 trials (24/48 = 50%). In the remaining trials, when the filler question was asked, we generated molecules for the question period that could lie on the current trajectory (34% of the time), or not (33% of the time it was out of trajectory by -60°, the other 33% of the time it was out of trajectory by +601. Given the complex and noncorresponding percentage of trials and expected responses, performance for this task was expressed as probability of Hits (probability of saying Yes when Yes was the correct answer) and False alarms (probability of saying Yes when No was the correct answer). Trials and egocentric-like conditions were also equally divided into the 4 quadrants composing the feature space, by controlling considering the midlines of both axes as boundaries.

Behavioral performance measures: Given the complex and noncorresponding percentage of trials and expected responses, performance for this task was expressed as probability of Hits (probability of saying Yes when Yes was the correct answer) and False alarms (probability of saying Yes when No was the correct answer).

Acquisition

Imaging type(s): functional

Field strength: 3T

Sequence & imaging parameters: High-resolution Tl-weighted images for anatomical localization were acquired at the end of the scan session using a three-dimensional pulse sequence with TR = 3.15 ms; TE = 1.37; flip angle = 8; voxel size = 1.6 x 1.6 x 1.6 mm). T2*-weighted images sensitive to blood oxygenation level-dependent (BOLD) contrasts were acquired using a pulse sequence with TR= 1500 ms; TE= 22 ms; flip angle = 80%voxel size = 2.5 x 2.5 x 2.5 mm; multiband acceleration factor of 3.

Area of acquisition: whole brain

Diffusion MRI: ☐ Used ☒ Not used

Preprocessing

Preprocessing software | Images were preprocessed using a standard pipeline with SPM12, which included slice-time correction, realignment of functional images to the middle scan of each run, coregistration of functional and anatomical images, segmentation, normalization to the Montreal Neurological Institute (MNI) space, and smoothing of 5 mm isotropic.

Normalization | standard normalization following the SPM12 manual

Normalization template | MNI

Noise and artifact removal | motion parameters were introduced as regressors to control for head motion

Volume censoring | NA

Statistical modeling & inference

Model type and settings | Adaptation and MVPa (decoding and RSA)

Effect(s) tested | We tested whether bold signal showed adaptation as a function of time since last presentation of a given egocentric-like condition
We tested if classifier was able to distinguish between egocentric like conditions in one context and across contexts
We used RSA and adaptation to look for 6-fold grid-like modulation of bold signal

Specify type of analysis: ☒ Whole brain ☐ ROI-based ☐ Both

Statistic type for inference | cluster wise

(See Eklund et al. 2016)

Correction | FWE correction, q <.05

Models & analysis

n/a	Involved in the study
☒	☐ Functional and/or effective connectivity
☒	☐ Graph analysis
☒	☐ Multivariate modeling or predictive analysis
